# Supplementary material for: Tree mortality during long-term droughts is lower in structurally complex forest stands
Source: Nat Commun. 2023 Nov 17;14:7467. doi: 10.1038/s41467-023-43083-8 (PMC10656564; doi:10.1038/s41467-023-43083-8)
Supplement: Supplementary file 1 — Supplementary Information [file 41467_2023_43083_MOESM1_ESM.pdf]

Supplementary Materials for

Overshadowing effects in structurally complex forests reduce tree mortality during drought

Qin Ma, Yanjun Su*, Chunyue Niu, Qin Ma, Tianyu Hu, Xiangzhong Luo, Xiaonan Tai, Tong Qiu, Yao Zhang, Roger C. Bales, Lingli Liu, Maggi Kelly, Qinghua Guo
*Corresponding author. Email: ysu@ibcas.ac.cn

This PDF file includes:
Supplementary Methods
Supplementary References
Supplementary Table 1. Accuracy of individual tree species classification.
Supplementary Table 2. Accuracy of dead tree detection.
Supplementary Table 3. Tree mortality rates of different drought periods within the study area.
Supplementary Table 4. Statistics of relationships between tree height and tree mortality rate during the 2012-2016 drought using forest stands as the basic statistical units.
Supplementary Table 5. Statistics of relationships between tree height and topographic wetness index (TWI).
Supplementary Table 6. Statistics of relationships between tree height and tree mortality rate during the 2012-2016 drought using regular grids as the basic statistical units.
Supplementary Table 7. Statistics of relationships between tree height and tree mortality rate during the 2012-2016 drought, considering the influence of the percentage threshold used to define dead trees.
Supplementary Table 8. Statistics of relationships between canopy cover taller than center tree height (CCTH) and tree mortality rate during the 2012-2016 drought using forest stands as the basic statistical units.
Supplementary Table 9. Statistics of relationships between CCTH and tree mortality rate during the 2012-2016 drought using regular grids as the basic statistical units.
Supplementary Table 10. Statistics of relationships between CCTH and tree mortality rate during the 2012-2016 drought, considering the influence of the percentage threshold used to define dead trees.
Supplementary Table 11. Statistics of relationships between CCTH and tree mortality rate during the 2012-2016 drought, considering the influence of the neighborhood size used to calculate CCTH.
Supplementary Table 12. Statistics of relationships between CCTH and tree mortality rate during the 2012-2016 drought, considering the confounding influence of tree height.
Supplementary Table 13. Comparisons of model explanation rates with or without considering canopy structural attributes.

Supplementary Table 14. The number of ground truth pixels for each of the six major land cover types within the study area for the years 2012, 2014, and 2016.
Supplementary Fig. 1 Tree species classification results over the study area in the southern Sierra Nevada mountains, California, USA.
Supplementary Fig. 2 Detected dead trees during the 2012-2014 drought over the study area.
Supplementary Fig. 3 Relationships between tree height and topographic wetness index (TWI) for all genera and each respective genus.
Supplementary Fig. 4 Relationships between canopy structure and tree mortality rate during the 2012-2016 drought using beta regression.
Supplementary Fig. 5 Relationships between canopy structure and tree mortality rate during the 2012-2016 drought using regular grids as the basic statistical units.
Supplementary Fig. 6 Sensitivity analysis of the relationships between canopy structure and tree mortality rate during the 2012-2016 drought, considering the influence of the percentage threshold used to define dead trees.
Supplementary Fig. 7 Sensitivity analysis of the relationships between CCTH and tree mortality rate during the 2012-2016 drought, considering the influence of the neighborhood size used to calculate CCTH.
Supplementary Fig. 8 A conceptual diagram illustrating the definitions of the three neighborhood canopy structural attributes used to quantify tree competition.
Supplementary Fig. 9 Relative importance of the three neighborhood canopy structural attributes to tree mortality rate during the 2012-2016 drought.
Supplementary Fig. 10 Responses of relative change in evapotranspiration (ET) to crown shadow ratio.
Supplementary Fig. 11 Pathways linking CCTH to tree mortality rate during drought through the regulation of crown shadow ratio derived from a structural equation modeling analysis.
Supplementary Fig. 12 Relationships of tree mortality rate during the 2012-2016 drought with CC66 and CVTH.
Supplementary Fig. 13 The distribution of field plot measurements and tie points within the study area.
Supplementary Fig. 14 Relationships between environmental factors and tree mortality rate during the 2012-2016 drought.
Supplementary Fig. 15 Relative importance of canopy structural attributes and environmental factors to tree mortality rate during the 2012-2016 drought.
Supplementary Fig. 16. Examples of segmented individual trees.
Supplementary Fig. 17 A conceptual diagram illustrating the simulation process of crown shadow.

Supplementary methods

Individual tree segmentation methods

We delineated individual tree crowns from the light detection and ranging (lidar) dataset collected in the summer of 2012 using a canopy height model (CHM)-based method. To generate a CHM covering the study area, we first preprocessed the collected lidar data following a procedure of outlier removal, filtering, and normalization (*1*), as detailed in the methods section. With the preprocessed lidar data, we interpolated a digital terrain model (DTM) and a digital surface model (DSM) from lidar ground points and lidar surface points, respectively. The CHM was then calculated as the difference between the DSM and the DTM. In this study, we utilized the ordinary kriging interpolation algorithm to generate both the DTM and the DSM (*2*), with a spatial resolution of 0.5 m (*3*).

The marker-controlled watershed segmentation algorithm was employed to segment individual tree crowns from the lidar-derived CHM. This algorithm combines the strengths of marker control, region growing, and edge detection, making it well-suited for tree segmentation in conifer-dominated forests (*3*). Here, the corresponding individual tree segmentation tool integrated within the LiDAR360 software (GreenValley International Inc.) was utilized for this task, and a non-tree mask with a threshold of 2 m (to remove areas with a height lower than 2 m), Gaussian smoothing (to fill pits in the CHM), and a searching radius of 5 ± 2 m were applied. Based on field measurements, tree segments with crown areas that were either too small (< 1 m^2) or too large (> 200 m^2) were likely attributed to noise or mis-segmentation. To mitigate their impact, we excluded all tree segments with crown areas smaller than 1 m^2, and manually divided tree segments with crown areas larger than 200 m^2 into smaller ones, if necessary. Subsequently, all identified individual tree crowns were manually reviewed and corrected for errors. In total, we delineated 1,405,237 individual tree crowns within the study area, of which 1,050,960 had a height greater than 5 m. Supplementary Fig. 16 presents two examples of segmented individual tree crowns along with their corresponding point clouds.

Individual tree species classification methods

Individual tree species were classified using a random forest classification approach. The ground truth for training the random forest classification model was provided by field-recorded individual tree species information. These field-recorded trees were matched with lidar-derived individual tree crowns based on their spatial locations, with tree height also considered a constraint, following the approach outlined in Ma et al. (*3*). Specifically, we began by generating a 2-m buffer around the location of each field-recorded tree and retained all lidar-segmented trees within the buffer as potential match candidates. Then, differences between field and lidar tree height measurements were assessed. If the height difference fell within the range of -1 m to 5 m, the corresponding lidar-derived tree segment was retained as a potential match candidate; otherwise, it was excluded. In cases where multiple potential match candidates persisted even after this refinement, the lidar-derived tree segment with the least disparity in tree height was selected as the final match. These tree matching steps accounted for potential positioning errors in field measurements and discrepancies between field-recorded and lidar-derived tree positions

(tree base *vs.* treetop). In total, 998 trees were measured in the field, and 977 of them could be matched with lidar-derived individual tree crowns, including 276 *Abies* tree, 425 *Cedrus* trees, 135 *Pinus* trees, and 141 *Quercus* trees. Half of these trees were randomly selected and used as training samples, while the remaining trees were used as validation samples to assess the accuracy of tree species classification.

A total of eight features were included in the random forest classification model, encompassing three lidar-derived canopy structural features (tree height, crown area, and ratio of tree height to crown area), one canopy spectral feature (normalized difference vegetation index/NDVI), and four topographic features (elevation, slope, aspect, and topographic wetness index/TWI). The NDVI of an individual tree was calculated as the average value of all National Agriculture Imagery Program (NAIP) pixels falling within its crown, with the pre-drought (2012) NAIP images used in this context. The four topographic features were calculated from the lidar-derived DTM, and their average values within each tree crown were employed.

The random forest classification model was implemented using R with the *randomForest* package, setting *mtry* (the number of features tried as each split) to 3 and the number of trees to 500. To further refine the classification results, we employed the altitudinal extent of each genus obtained from the vegetation map of Classification and Assessment with Landsat of Visible Ecological Groupings system as constraints (*4*). If a classified individual tree fell out of its corresponding altitudinal range, we reclassified it using samples of trees belonging to that specific altitudinal range.

The individual tree species classification results are presented in Supplementary Fig. 1. The overall accuracy was 66%, and the kappa coefficient was 0.5 (Supplementary Table 1). The highest user accuracy was observed for *Cedrus* (76%), followed by *Quercus* (67%), *Abies* (59%), and *Pinus* (48%); the highest producer accuracy was observed for *Abies* (77%), followed by *Cedrus* (73%), *Quercus* (53%), and *Pinus* (34%) (Supplementary Table 1).

Dead tree detection methods

Dead tree detection serves as the foundation step in calculating tree mortality rate. In this study, the identification of dead trees relied on time-series National Agriculture Imagery Program (NAIP) imagery, using lidar-derived individual tree crowns as a basis. Specifically, NAIP imagery from 2012, 2014, and 2016, corresponding to pre-drought, early-drought, and peak-drought conditions, respectively, were employed. The processing procedure applied to the NAIP imagery of each year remained consistent, which is elaborated upon as follows.

To detect dead trees, we first recognized six major land cover types within the study area, which were dead tree, green tree, grass, bare ground, artificial object, and water. Then, a total of 350 land patches with homogenous land cover, encompassing 61,965 NAIP pixels, were randomly delineated and visually interpreted by experienced researchers. These pixels were utilized as the ground truth for the dead tree detection procedure. Supplementary Table 14 shows the number of pixels for each land cover type.

Random forest classification was employed to categorize the study area into the aforementioned six land cover types at the pixel level. The four spectral bands and NDVI were utilized as inputs for the classification. Half of the ground truth pixels were randomly selected to train the random forest model, which was implemented using the *randomForest* package in R,

with *mtry* and the number of trees set to 3 and 500. The pixel-level classification results were further overlaid with the lidar-derived tree crowns. If over 35% of an individual tree crown was classified as dead tree, it was identified as such, following the suggestion of Stovall et al. (*5*). If a tree was identified as dead in the pre-drought aerial imagery, it was not considered a dead tree that occurred during the drought and was subsequently removed from the early-drought and peak-drought dead tree detection results.

To assess the accuracy of the dead tree detection results, the remaining half of the visually interpreted pixels were employed to determine the dead/live conditions of 635 trees. Similar to the approach mentioned above, if 35% of a tree's crown was covered by dead tree pixels, it was identified as a dead tree; otherwise, it was identified as a live tree. Following this procedure, 2, 22, and 113 out of the 635 trees were identified as dead in 2012, 2014, and 2016, respectively. For each genus and drought period (pre-drought, early-drought, and peak-drought), the recall, precision, and F-score were computed to quantify the dead tree detection accuracy (*6*).

The overall dead tree detection accuracy, quantified by F-score was 0.67, 0.9, and 0.91 for 2012, 2014 and 2016, respectively. Among the four genera, *Cedrus* exhibited the highest dead tree detection accuracy, followed by *Abies*, *Quercus*, and *Pinus* (Supplementary Table 2). The final dead tree detection results for 2014 and 2016 are presented in Supplementary Fig. 2.

It is important to note that the dead tree detection results may vary based on the percentage threshold used to determine dead trees. To ensure the robustness of the subsequent statistical analyses, we repeated the dead tree detection process by changing the percentage threshold from 30% to 50% in 5% increments. Each of the dead tree detection results was then used to evaluate the relationships between canopy structure and tree mortality rate, respectively.

Crown shadow and evapotranspiration simulation methods

The shaded canopy of each tree caused by its neighboring trees was simulated using a ray tracing method (*7*). To achieve this, we first voxelized the lidar point cloud within the neighborhood of a tree at a spatial resolution of 1 m × 1 m × 1 m (Supplementary Fig. 17a and b). Here, the neighborhood of a tree was defined as a circular buffer of 15 m in radius, which was approximately three times of the average crown radius in the study area. Voxels with lidar points were labeled as nonempty voxels, while others were labeled as empty voxels. From the center of each nonempty voxel at the crown surface, a ray was generated towards the direction of the sun. If the ray was blocked before reaching the sun, it was recorded as a shaded voxel; otherwise, it was recorded as a sunlit voxel (Supplementary Fig. 17c). This process was repeated 23 times for each tree (simulated once every 30 minutes from 7 am to 6:30 pm) for a typical summer day in the study area (August 1st, 2016). The crown shadow ratio of a tree at each time stamp was calculated as the ratio of the number of shaded voxels to the number of canopy voxels at the crown surface. The final crown shadow ratio during the daytime was calculated as the average crown shadow ratio of all time stamps, weighted by the solar radiation at each time stamp. The solar radiation of a tree at each time stamp was calculated from the lidar-derived DTM using the Area Solar Radiation tool in ArcGIS (ESRI).

Changes in crown shadow ratio can influence the evapotranspiration (ET) of a tree and, consequently, its survival rate during drought (*8*). To comprehend the impact of crown shadow ratio on ET, we simulated the relative changes in ET under various crown shadow ratios using an established terrestrial biosphere model, the Boreal Ecosystem Productivity Simulator (BEPS) (*9*). BEPS is a two-leaf enzyme kinetic model capable of simulating water-carbon fluxes in a coupled manner (*9*), and its ET estimates have been extensively validated across global eddy covariance sites (*10, 11*).

In BEPS, the ET of a tree is calculated as follows,

$$ET = ET_{sun} + ET_{sh} \quad \text{(Eq. 1)}$$

where ET_{sun} refers to the ET from the sunlit crown, and ET_{sh} refers to the ET from the shaded crown. From Eq. 1, we can further have,

$$EET_{sun} = LAI_{sun} \times Tran_{sun} \quad \text{(Eq. 2)}$$

$$ET_{sh} = LAI_{sh} \times Tran_{sh} \quad \text{(Eq. 3)}$$

where LAI_{sun} and LAI_{sh} represent the leaf area index (LAI) of the sunlit crown and the shaded crown, and $Tran_{sun}$ and $Tran_{sh}$ represent the leaf transpiration of the sunlit crown and the shaded crown, respectively. Assuming a condition in which an individual tree has no shaded crown, its ET (ET_{ref}) is completely contributed by the sunlit crown. In this study, the relative change in ET was calculated as the ratio between ET of a tree under various crown shadow ratios (ranging from 73% to 92% with an interval of 3%) to that of a tree without crown shadow, which can be calculated as follows,

$$\begin{aligned} ET/\mathrm{ET}_{ref} &= (LAI_{sun} \times Tran_{sun} + LAI_{sh} \times Tran_{sh})/(LAI \times Tran_{sun}) \\ &= LAI_{sun}/LAI + (1 - LAI_{sun}/LAI) \times (Tran_{sh}/Tran_{sun}) \end{aligned} \quad \text{(Eq. 4)}$$

where LAI is the total LAI, equaling to $LAI_{sun}+LAI_{sh}$.

In this study, we utilized both a conifer-shaped tree and a sphere-shaped tree as examples to simulate the relative ET change under different crown shadow ratios. LAI_{sun} and LAI_{sh} under different crown shadow ratios were estimated from the simulated shaded crown and sunlit crown using a voxel-based method, as described in Li et al. (*12*). $Tran_{sh}$ and $Tran_{sun}$ for a typical summer day in the study area (August 1st, 2016) were simulated using the BEPS model. The corresponding climate and biophysical conditions of the model were derived from the meteorological records of an eddy covariance site (US-Ton) near the study area (*13*). A clumping index of 0.8 was applied in the model (*9*).

Supplementary references

1. Q. Guo *et al.*, Lidar Boosts 3D Ecological Observations and Modelings: A Review and Perspective. *IEEE Geosci. Remote Sens. Mag.* **9**, 232-257 (2021).
2. Q. Guo, W. Li, H. Yu, O. Alvarez, Effects of topographic variability and lidar sampling density on several DEM interpolation methods. *Photogramm. Eng. Remote Sens.* **76**, 701-712 (2010).
3. Q. Ma, Y. Su, S. Tao, Q. Guo, Quantifying individual tree growth and tree competition using bi-temporal airborne laser scanning data: a case study in the Sierra Nevada Mountains, California. *Int. J. Digit. Earth* **11**, 1-19 (2017).
4. J. Franklin, C. E. Woodcock, R. Warbington, Multi-attribute vegetation maps of forest service lands in California supporting resource management decisions. *Photogramm. Eng. Remote Sens.* **66**, 1209-1218 (2000).
5. A. E. L. Stovall, H. Shugart, X. Yang, Tree height explains mortality risk during an intense drought. *Nat. Commun.* **10**, 4385 (2019).
6. W. Li, Q. Guo, A New Accuracy Assessment Method for One-Class Remote Sensing Classification. *IEEE Trans. Geosci. Remote Sensing,* **52**, 4621-4632 (2014).
7. D. Kükenbrink, F. D. Schneider, R. Leiterer, M. E. Schaepman, F. Morsdorf, Quantification of hidden canopy volume of airborne laser scanning data using a voxel traversal algorithm. *Remote Sens. Environ.* **194**, 424-436 (2017).
8. W. R. L. Anderegg, J. A. Berry, C. B. Field, Linking definitions, mechanisms, and modeling of drought-induced tree death. *Trends Plant Sci.* **17**, 693-700 (2012).

9. J. M. Chen, J. Liu, J. Cihlar, M. L. Goulden, Daily canopy photosynthesis model through temporal and spatial scaling for remote sensing applications. *Ecol. Model.* **124**, 99-119 (1999).
10. L. He *et al.*, Changes in the Shadow: The Shifting Role of Shaded Leaves in Global Carbon and Water Cycles Under Climate Change. *Geophys. Res. Lett.* **45**, 5052-5061 (2018).
11. X. Luo *et al.*, Comparison of Big‐Leaf, Two‐Big‐Leaf, and Two‐Leaf Upscaling Schemes for Evapotranspiration Estimation Using Coupled Carbon‐Water Modeling. *J. Geophys. Res.-Biogeosci.* **123**, 207-225 (2018).
12. Y. Li *et al.*, Derivation, validation, and sensitivity analysis of terrestrial laser scanning-based leaf area index. *Can. J. Remote Sens.* **42**, 719-729 (2016).
13. S. Ma, D. Baldocchi, S. Wolf, J. Verfaillie, Slow ecosystem responses conditionally regulate annual carbon balance over 15 years in Californian oak-grass savanna. *Agric. For. Meteorol.* **228-229**, 252-264 (2016).

Supplementary Table 1. Accuracy of individual tree species classification. The accuracy of each genus was assessed using independent field measurements (see Supplementary Methods for details). Note that OA and *k* represents overall accuracy and kappa coefficient, respectively.

	Abies	*Cedrus*	*Pinus*	*Quercus*	Precision
Abies	117	21	11	2	77%
Cedrus	30	171	15	17	73%
Pinus	48	0	25	1	34%
Quercus	2	33	1	41	53%
Recall	59%	76%	48%	67%	OA=66%, *k*=0.50

Supplementary Table 2. Accuracy of dead tree detection. Note that the years 2012, 2014, and 2016 represent the dead tree detection results using National Agriculture Imagery Program imagery from those respective years, and a percentage threshold of 35% were used to present the tree mortality rates within the study area (see Supplementary Methods for details). *All* represents all genera, F represents F-score, and NA means the corresponding information is not available.

**Genus**	**2012**			**2014**			**2016**		
	Recall	Precision	F	Recall	Precision	F	Recall	Precision	F
All	50%	100%	0.67	91%	88%	0.90	91%	93%	0.92
Abies	100%	100%	1	88%	88%	0.88	90%	93%	0.91
Cedrus	NA	0%	NA	91%	84%	0.87	92%	93%	0.92
Pinus	0%	NA	NA	100%	60%	0.75	100%	89%	0.94
Quercus	NA	NA	NA	71%	100%	0.83	87%	100%	0.93

Supplementary Table 3. Tree mortality rates of different drought periods within the study area. The entire drought period was divided into two periods, i.e., early drought (2012-2014) and peak drought (2014-2016). The tree mortality rate was calculated from dead tree detection results using a percentage threshold varying from 30% to 50% with a 5% increment (see Supplementary Methods for details). The dead tree detection results from a percentage threshold of 35% were used to present the tree mortality rates within the study area, while the results from other percentage thresholds were used to validate the relationships between canopy structure and tree mortality rate (see Supplementary Methods for details).

Genus	Tree mortality rates calculated from varying percentage thresholds defining dead trees														
	**30%**			**35%**			**40%**			**45%**			**50%**		
	2012 | 2014	2014 | 2016	2012 | 2016	2012 | 2014	2014 | 2016	2012 | 2016	2012 | 2014	2014 | 2016	2012 | 2016	2012 | 2014	2014 | 2016	2012 | 2016	2012 | 2014	2014 | 2016	2012 | 2016
All	0.31	8.99	6.06	0.09	8.60	4.35	0.04	6.22	3.13	0.04	4.83	2.43	0.03	3.76	1.90
Abies	0.24	11.19	6.82	0.12	10.34	5.23	0.06	8.20	4.13	0.06	6.27	3.16	0.05	4.82	2.43
Cedrus	0.26	5.28	3.96	0.06	4.95	2.51	0.03	3.90	1.96	0.02	2.99	1.51	0.02	2.32	1.17
Pinus	0.24	10.71	6.53	0.09	10.41	5.25	0.04	6.23	3.13	0.03	4.88	2.46	0.03	3.86	1.94
Quercus	0.79	5.13	6.51	0.08	5.39	2.73	0.02	4.55	2.29	0.02	3.69	1.85	0.01	2.91	1.46

Supplementary Table 4. Statistics of relationships between tree height and tree mortality rate during the 2012-2016 drought using forest stands as the basic statistical units. Relationships between tree height and tree mortality rate were evaluated using both weighted piecewise linear regression and beta regression. Tree mortality rate within each forest stand was binned by tree height with an interval of 2 m. Start and End represent the starting and ending tree height of each segment, and R^2 and P represent coefficient of determination and p-value. The slope in beta regression was the regression coefficient of tree height after log transformation.

Genus	Segments		Linear regression			Beta regression		
	Start, m	End, m	Slope	R^2	P	Slope	R^2	P
All	5	14	-0.178	0.98	<0.001	-0.046	0.98	<0.001
	14	39	0.104	0.96	<0.001	0.024	0.94	<0.001
	39	60	-0.064	0.67	<0.001	-0.014	0.64	<0.001
Abies	5	27	-0.085	0.72	0.025	-0.016	0.74	<0.001
	27	40	0.047	0.88	<0.001	0.010	0.88	<0.001
	40	60	-0.041	0.49	<0.001	-0.009	0.48	<0.001
Cedrus	5	17	-0.041	0.76	<0.001	-0.017	0.77	<0.001
	17	29	0.067	0.90	<0.001	0.028	0.90	<0.001
	29	38	-0.062	0.22	<0.001	-0.024	0.22	<0.001
Pinus	5	15	-0.136	0.88	<0.001	-0.029	0.90	<0.001
	15	33	0.133	0.96	<0.001	0.026	0.96	<0.001
	33	60	-0.123	0.85	<0.001	-0.024	0.81	<0.001
Quercus	5	14	-0.329	0.98	<0.001	-0.112	0.98	<0.001
	14	25	0.130	0.92	<0.001	0.057	0.92	<0.001

Supplementary Table 5. Statistics of relationships between tree height and topographic wetness index (TWI). Relationships between tree height and TWI were simulated using weighted piecewise linear regression, using forest stands as the basic statistical units. TWI within each forest stand was binned by tree height with an interval of 2 m, and the piecewise linear regression method weighted by the number of trees in each bin was used to fit their relationships.

**Genus**	**Segments**		**Model statistics**		
	Start, m	End, m	Slope	R^2	P
All	5	34	0.002	0.35	0.015
	34	60	0.014	0.92	<0.001
Abies	5	34	-0.01	0.83	<0.001
	34	60	0.012	0.92	<0.001
Cedrus	5	20	-0.002	0.52	0.028
	20	38	0.02	0.81	<0.001
Pinus	5	32	-0.002	0.23	0.069
	32	60	0.017	0.71	<0.001
Quercus	5	25	0.005	0.81	<0.001

Supplementary Table 6. Statistics of relationships between tree height and tree mortality rate during the 2012-2016 drought using regular grids as the basic statistical units. Relationships between tree height and tree mortality rate were simulated using weighted piecewise linear regression. Tree mortality rate within each grid cell (500 m × 500 m) was binned by tree height with an interval of 2 m, and the piecewise linear regression method weighted by the number of trees in each bin was used to fit their relationships.

**Genus**	**Segments**		**Model statistics**		
	Start, m	End, m	Slope	R^2	*P*
All	5	14	-0.065	0.88	<0.001
	14	32	0.174	0.96	<0.001
	32	60	-0.119	0.79	<0.001
Abies	5	14	-0.077	0.37	0.025
	14	32	0.111	0.98	<0.001
	32	60	-0.059	0.35	<0.001
Cedrus	5	14	-0.101	0.77	0.050
	14	28	0.141	0.96	<0.001
	28	38	-0.115	0.71	<0.001
Pinus	5	14	-0.172	0.86	<0.001
	14	32	0.178	0.96	<0.001
	32	60	-0.137	0.77	<0.001
Quercus	5	14	-0.09	0.94	<0.001
	14	25	0.214	0.94	<0.001

Supplementary Table 7. Statistics of relationships between tree height and tree mortality rate during the 2012-2016 drought, considering the influence of the percentage threshold used to define dead trees. Tree mortality rate were determined by a percentage threshold varying from 30% to 50% with an interval of 5%. Forest stands were used as the basic statistical units here. Tree mortality rate within each forest stand was binned by tree height with an interval of 2 m, and the piecewise linear regression method weighted by the number of trees in each bin was used to fit their relationships.

Percentage threshold	Segments		Model statistics		
	Start, m	End, m	Slope	R^2	P
30%	5	14	-0.187	0.92	<0.001
	14	32	0.164	0.98	<0.001
	32	60	-0.130	0.94	<0.001
35%	5	14	-0.178	0.98	<0.001
	14	32	0.104	0.96	<0.001
	32	60	-0.064	0.67	<0.001
40%	5	14	-0.058	0.72	<0.001
	14	32	0.127	0.97	<0.001
	32	60	-0.074	0.80	<0.001
45%	5	14	-0.054	0.75	<0.001
	14	32	0.097	0.96	<0.001
	32	60	-0.061	0.78	<0.001
50%	5	14	-0.044	0.74	<0.001
	14	32	0.075	0.95	<0.001
	32	60	-0.049	0.72	<0.001

Supplementary Table 8. Statistics of relationships between canopy cover taller than center tree height (CCTH) and tree mortality rate during the 2012-2016 drought using forest stands as the basic statistical units. Relationships between CCTH and tree mortality rate were simulated using both weighted linear regression and beta regression. Tree mortality rate within each forest stand was binned by CCTH with an interval of 4%. The slope in beta regression was the regression coefficient of CCTH after log transformation.

**Genus**	**Linear regression**			**Beta regression**		
	Slope	R^2	P	Slope	R^2	P
All	-0.017	0.86	<0.001	-0.363	0.85	<0.001
Abies	-0.009	0.42	<0.001	-0.185	0.55	<0.001
Cedrus	-0.007	0.34	<0.001	-0.312	0.59	<0.001
Pinus	-0.018	0.76	<0.001	-0.265	0.90	<0.001
Quercus	-0.010	0.31	<0.001	-0.466	0.72	<0.001

Supplementary Table 9. Statistics of relationships between CCTH and tree mortality rate during the 2012-2016 drought using regular grids as the basic statistical units. Relationships between CCTH and tree mortality rate were simulated using weighted linear regression. Tree mortality rate within each grid cell (500 m × 500 m) was binned by CCTH an interval of 4%.

**Genus**	**Model statistics**		
	Slope	R^2	P
All	-0.009	0.90	<0.001
Abies	-0.007	0.58	<0.001
Cedrus	-0.006	0.52	<0.001
Pinus	-0.012	0.64	<0.001
Quercus	-0.009	0.41	<0.001

Supplementary Table 10. Statistics of relationships between CCTH and tree mortality rate during the 2012-2016 drought, considering the influence of the percentage threshold used to define dead trees. Tree mortality rate were determined by a percentage threshold varying from 30% to 50% with an interval of 5%. Forest stands were used as the basic statistical units here. Tree mortality rate within each forest stand was binned by CCTH with an interval of 4%, and the linear regression method weighted by the number of trees in each bin was used to fit their relationships.

**Percentage threshold**	**Model statistics**		
	Slope	R^2	P
30%	-0.017	0.92	<0.001
35%	-0.017	0.86	<0.001
40%	-0.009	0.87	<0.001
45%	-0.005	0.79	<0.001
50%	-0.002	0.47	<0.001

Supplementary Table 11. Statistics of relationships between CCTH and tree mortality rate during the 2012-2016 drought, considering the influence of the neighborhood size used to calculate CCTH. CCTH was calculated using four different neighborhood sizes, with radii of 15 m, 30 m, 50 m, and 100 m, respectively. Forest stands were used as the basic statistical units here. Tree mortality rate within each forest stand was binned by CCTH with an interval of 4%, and the linear regression method weighted by the number of trees in each bin was used to fit their relationships.

**Genus**	**Neighborhood Size (m)**	**Model statistics**		
		Slope	R^2	P
All	15	-0.017	0.86	<0.001
	30	-0.118	0.86	<0.001
	50	-0.112	0.99	<0.001
	100	-0.118	0.98	<0.001
Abies	15	-0.009	0.42	<0.001
	30	-0.123	0.46	<0.001
	50	-0.077	0.90	<0.001
	100	-0.083	0.95	<0.001
Cedrus	15	-0.007	0.34	<0.001
	30	-0.127	0.93	<0.001
	50	-0.120	0.95	<0.001
	100	-0.127	0.90	<0.001
Pinus	15	-0.018	0.76	<0.001
	30	-0.134	0.97	<0.001
	50	-0.136	0.98	<0.001
	100	-0.138	0.92	<0.001
Quercus	15	-0.010	0.31	<0.001
	30	-0.091	0.93	<0.001
	50	-0.094	0.95	<0.001
	100	-0.102	1.00	<0.001

Supplementary Table 12. Statistics of relationships between CCTH and tree mortality rate during the 2012-2016 drought, considering the confounding influence of tree height. Tree height was used as both a categorical random effect and a continuous random effect in the mixed linear modeling analyses. For the categorical random effect, tree height was divided into three groups, which were < 33rd percentile, 33rd percentile - 66th percentile, and > 66th percentile. Tree mortality rate within each forest stand was binned by CCTH an interval of 4%. The slope and significance (represented by *P*) of the fixed effect (CCTH) and the significance (represented by *P*) of the random effect (tree height group) were reported. The significance of the random effect was determined by comparing the mixed linear effect model for all genera or each genus with its corresponding model that did not account for tree height as the random effect, using analysis of variance.

**Genus**	**Fixed effect: CCTH**		**Random effect: Tree height**
	**Slope**	***P***	***P***
Using tree height as a categorical random effect			
All	-0.014	<0.001	<0.001
Abies	-0.010	<0.001	<0.001
Cedrus	-0.007	<0.001	<0.001
Pinus	-0.012	<0.001	<0.001
Quercus	-0.014	<0.001	<0.001
Using tree height as a continuous random effect			
All	-0.007	<0.001	<0.001
Abies	-0.011	<0.001	<0.001
Cedrus	-0.005	<0.001	<0.001
Pinus	-0.009	<0.001	<0.001
Quercus	-0.014	<0.001	<0.001

Supplementary Table 13. Comparisons of model explanation rates with or without considering canopy structural attributes. Each model was built using the random forest regression method. TH and Solar represent tree height and solar radiation. Both the model training R^2 (R^2_{train}) and the validation R^2 ($R^2_{validation}$) using independent samples are reported.

Model	Genus	R^2_{train}	$R^2_{validation}$
Mortality ~ CCTH+TH+Elevation+TWI+Solar	*All*	0.73	0.64
Mortality ~ Elevation+TWI+Solar	*All*	0.54	0.51
Mortality ~ CCTH+TH+Elevation+TWI+Solar	*Abies*	0.74	0.67
Mortality ~ Elevation+TWI+Solar	*Abies*	0.59	0.6
Mortality ~ CCTH+TH+Elevation+TWI+Solar	*Cedrus*	0.57	0.42
Mortality ~ Elevation+TWI+Solar	*Cedrus*	0.37	0.36
Mortality ~ CCTH+TH+Elevation+TWI+Solar	*Pinus*	0.63	0.44
Mortality ~ Elevation+TWI+Solar	*Pinus*	0.4	0.33
Mortality ~ CCTH+TH+Elevation+TWI+Solar	*Quercus*	0.64	0.48
Mortality ~ Elevation+TWI+Solar	*Quercus*	0.41	0.38

Supplementary Table 14. The number of ground truth pixels for each of the six major land cover types within the study area for the years 2012, 2014, and 2016. These ground truth pixels were obtained from 350 randomly selected land patches with homogeneous land cover and were visually interpreted by experienced researchers.

Year	Water	Artificial object	Bare ground	Grass	Green tree	Dead tree	Total
2012	16794	4623	11939	143	28243	366	61965
2014	16793	4629	11730	143	25943	2870	61965
2016	16676	4690	11988	143	13113	15498	61965

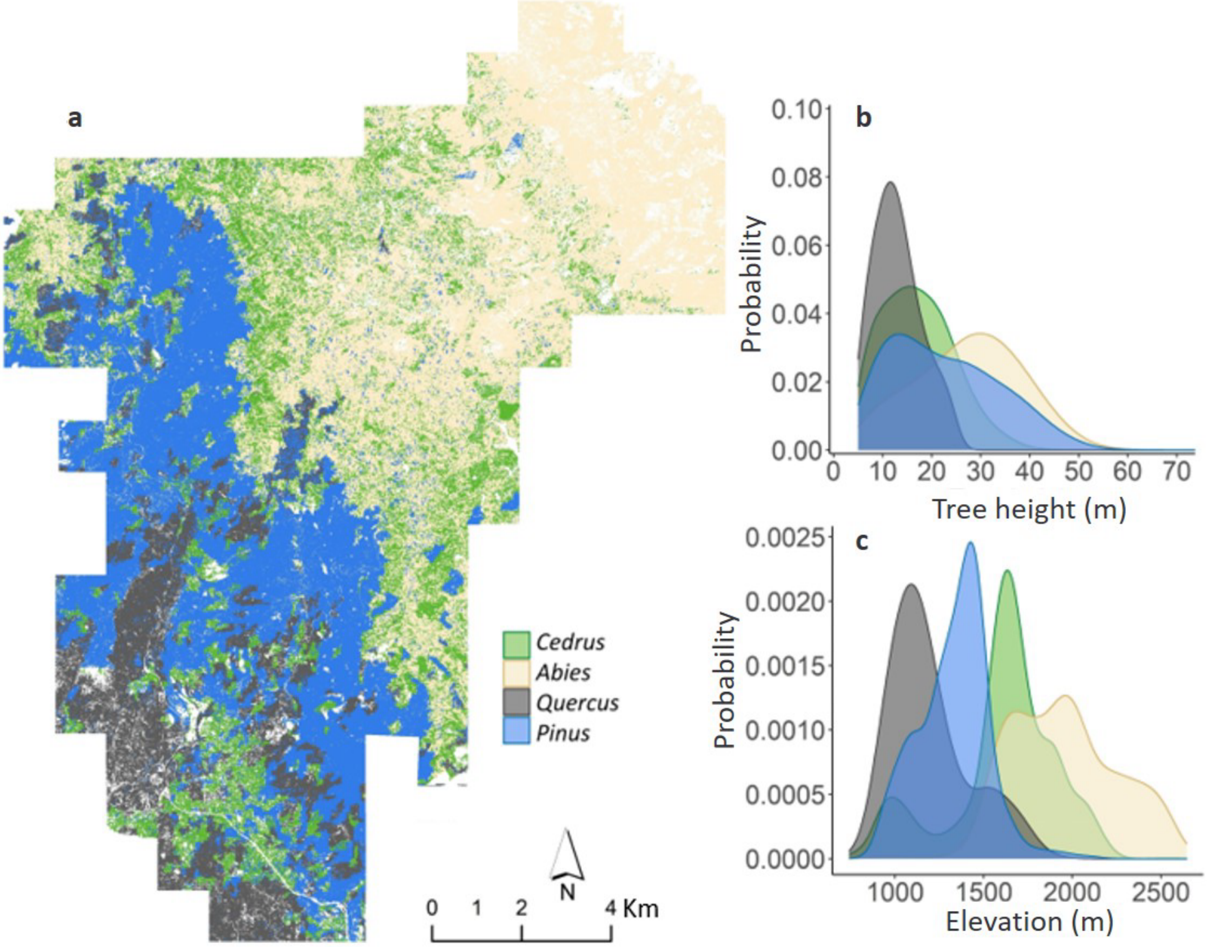

Supplementary Fig. 1 Tree species classification results over the study area in the southern Sierra Nevada mountains, California, USA. a, The spatial distribution of the four classified genera, i.e., *Abies, Cedrus, Quercus,* and *Pinus*. **b,** Histogram of tree height for the four genera. **c,** Histogram of elevation for the four genera.

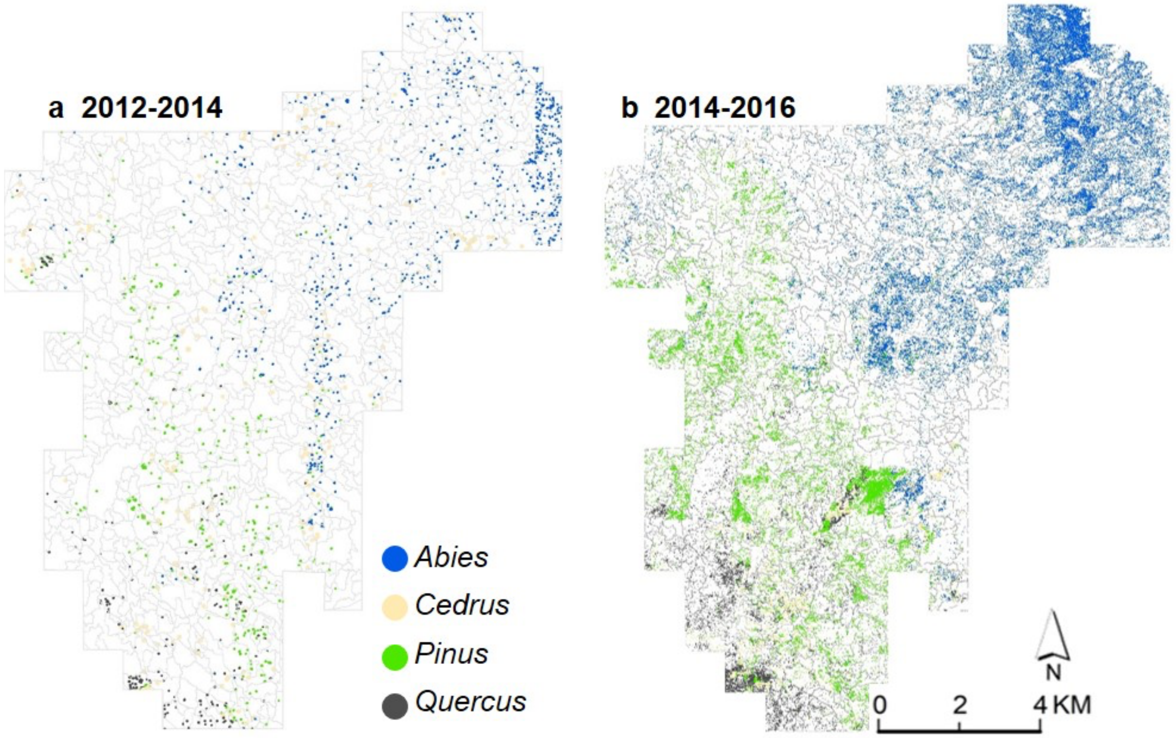

Supplementary Fig. 2 Detected dead trees during the 2012-2014 drought over the study area. a, Map of the detected dead trees for the year 2014. **b,** Map of the detected dead trees for the year 2016.

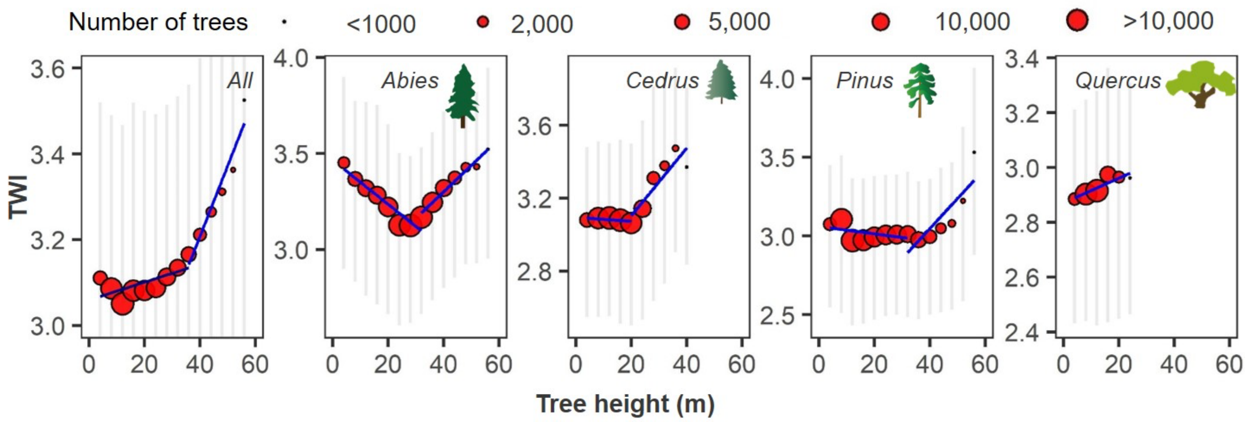

Supplementary Fig. 3 Relationships between tree height and topographic wetness index (TWI) for all genera and each respective genus. Forest stands were used as the basic statistical units here. TWI within each forest stand was binned by tree height with an interval of 2 m, and the piecewise linear regression method weighted by the number of trees in each bin was used to fit their relationships. Red dots in all panels represent the average TWI within each tree height bin, and grey lines represent the range of TWI between the first and third quartiles of each bin. Only half of the bins were presented for visual clarity. The size of red dots is proportional to the number of trees in each bin. Solid blue lines are fitted lines with a *p*-value (*P*) < 0.1. The corresponding statistics of each regression segment are presented in Supplementary Table 5.

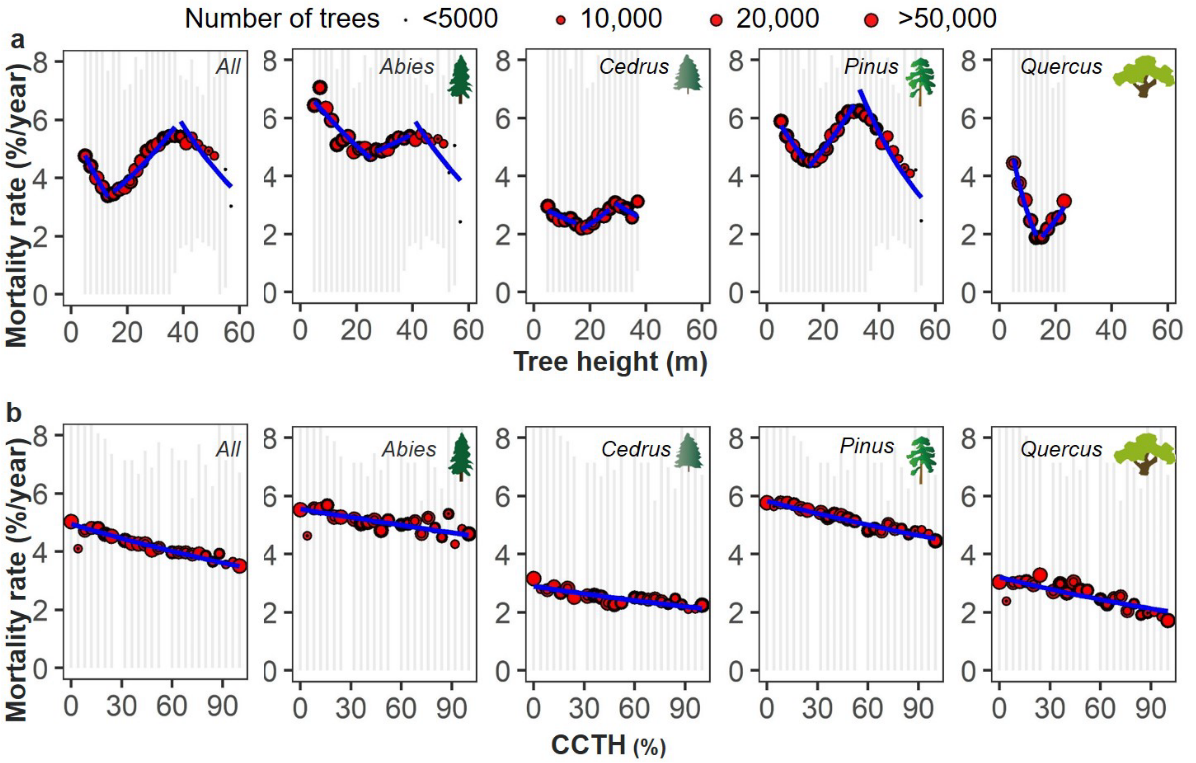

Supplementary Fig. 4 Relationships between canopy structure and tree mortality rate during the 2012-2016 drought using beta regression. a, Relationships between tree height and tree mortality rate of all genera (represented by panel *All*) and each respective genus, using forest stands as the basic statistical units. Tree mortality rate within each forest stand was binned by tree height with an interval of 2 m. **b,** Relationships between canopy cover taller than center tree height (CCTH) and tree mortality rate of all genera (represented by panel *All*) and each respective genus, using forest stands as the basic statistical units. Tree mortality rate within each forest stand was binned by CCTH with an interval of 4%. Red dots in all panels represent the average tree mortality within each tree height or CCTH bin, and grey lines represent the range of tree mortality rate between the first and third quartiles of each bin. Only half of the bins were presented for visual clarity. The size of red dots is proportional to the number of trees in each bin. Solid blue lines are fitted lines with a $P < 0.05$. The corresponding statistics of each regression segment are presented in Supplementary Tables 4 and 8.

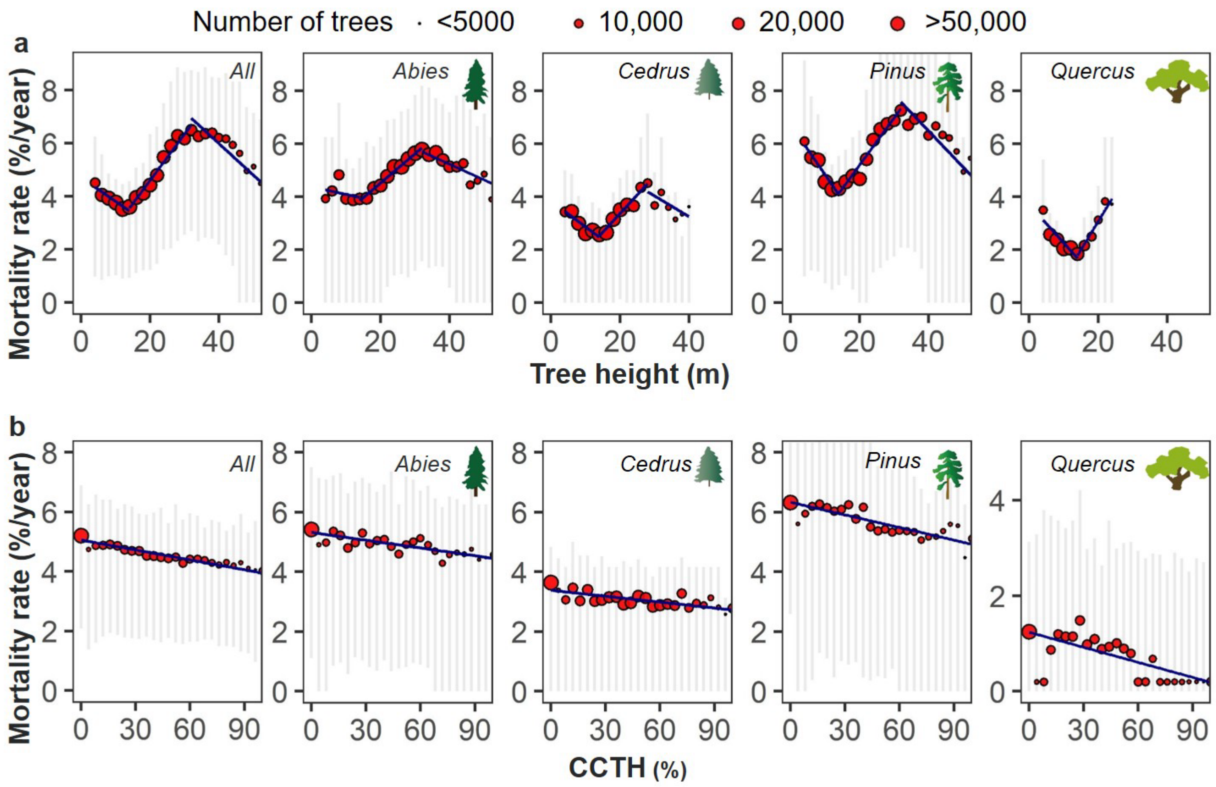

Supplementary Fig. 5 Relationships between canopy structure and tree mortality rate during the 2012-2016 drought using regular grids as the basic statistical units. a, Relationships between tree height and tree mortality rate of all genera (represented by panel *All*) and each respective genus. Tree mortality rate within each grid cell (500 m × 500 m) was binned by tree height with an interval of 2 m, and the piecewise linear regression method weighted by the number of trees in each bin was used to fit their relationships. **b,** Relationships between CCTH and tree mortality rate of all genera (represented by panel *All*) and each respective genus. Tree mortality rate within each grid cell (500 m × 500 m) was binned by CCTH with an interval of 4%, and the linear regression method weighted by the number of trees in each bin was used to fit their relationships. Red dots in all panels represent the average tree mortality within each tree height or CCTH bin, and grey lines represent the range of tree mortality rate between the first and third quartiles of each bin. Only half of the bins were presented for visual clarity. The size of red dots is proportional to the number of trees in each bin. Solid blue lines are fitted lines with a $P < 0.05$. The corresponding statistics of each regression segment are presented in Supplementary Tables 6 and 9.

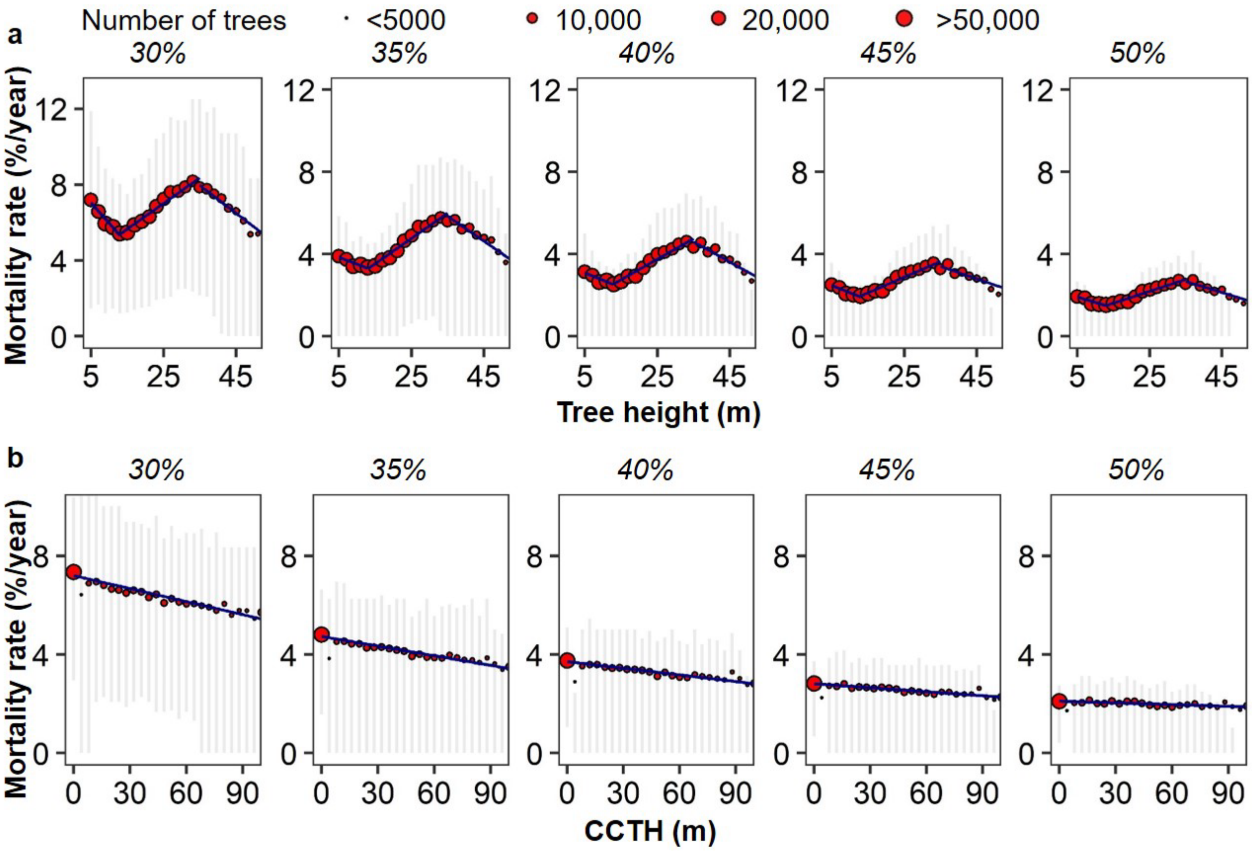

Supplementary Fig. 6 Sensitivity analysis of the relationships between canopy structure and tree mortality rate during the 2012-2016 drought, considering the influence of the percentage threshold used to define dead trees. a, Relationships between tree height and tree mortality rate determined by a percentage threshold varying from 30% to 50% with an interval of 5%. Forest stands were used as the basic statistical units here. Tree mortality rate within each forest stand was binned by tree height with an interval of 2 m, and the piecewise linear regression method weighted by the number of trees in each bin was used to fit their relationships. **b,** Relationships between CCTH and tree mortality rate determined by a percentage threshold varying from 30% to 50% with an interval of 5%. Forest stands were used as the basic statistical units here. Tree mortality rate within each forest stand was binned by CCTH with an interval of 4%, and the linear regression method weighted by the number of trees in each bin was used to fit their relationships. Red dots in all panels represent the average tree mortality within each tree height or CCTH bin, and grey lines represent the range of tree mortality rate between the first and third quartiles of each bin. Only half of the bins were presented for visual clarity. The size of red dots is proportional to the number of trees in each bin. Solid blue lines are fitted lines with a $P < 0.05$. The corresponding statistics of each regression segment are presented in Supplementary Tables 7 and 10.

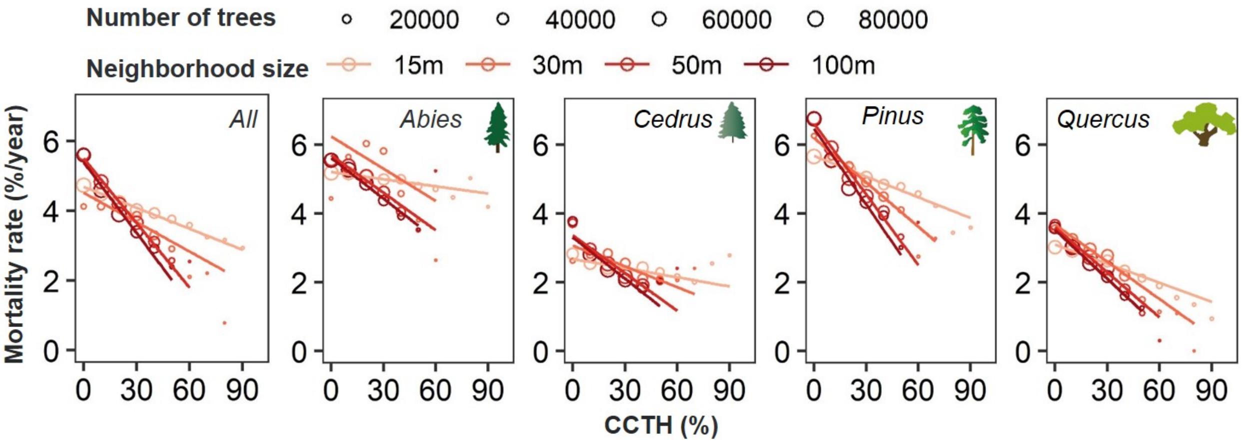

Supplementary Fig. 7 Sensitivity analysis of the relationships between CCTH and tree mortality rate during the 2012-2016 drought, considering the influence of the neighborhood size used to calculate CCTH. CCTH was calculated using four different neighborhood sizes, with radii of 15 m, 30 m, 50 m, and 100 m, respectively. Forest stands were used as the basic statistical units here. Tree mortality rate within each forest stand was binned by CCTH with an interval of 10%, and the linear regression method weighted by the number of trees in each bin was used to fit their relationships. Red dots in all panels represent the average tree mortality within each tree height or CCTH bin. Only half of the bins were presented for visual clarity. The size of red dots is proportional to the number of trees in each bin. Solid lines are fitted lines with a $P < 0.05$. The corresponding statistics of each regression segments are presented in Supplementary Table 11.

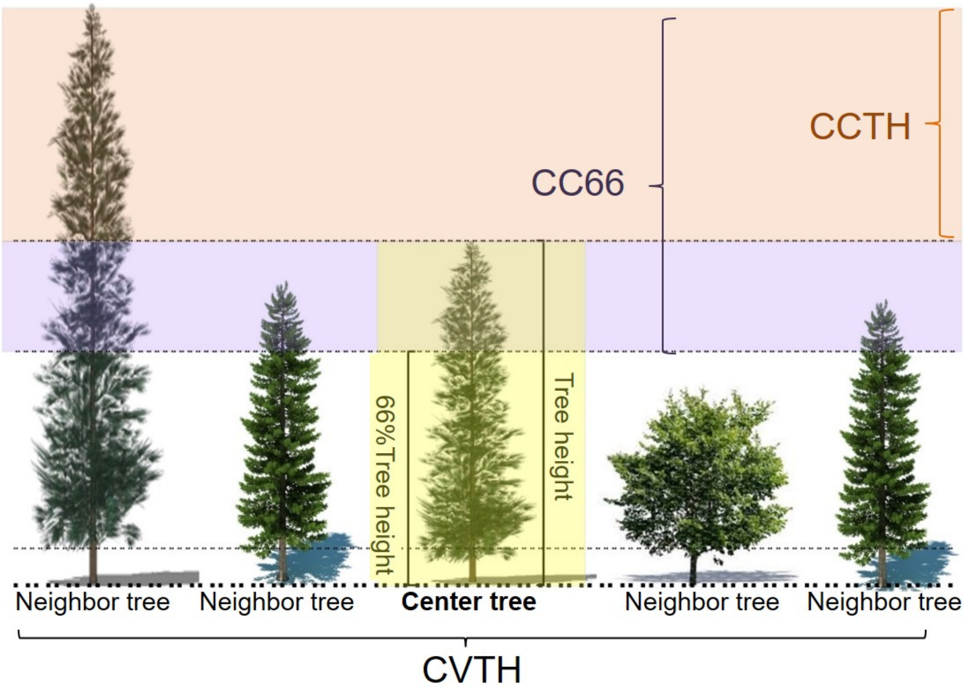

Supplementary Fig. 8 A conceptual diagram illustrating the definitions of the three neighborhood canopy structural attributes used to quantify tree competition. CCTH, CC66, and CVTH represent canopy cover taller than center tree height, canopy cover taller than 66% center tree height, and coefficient of variation in tree height, respectively.

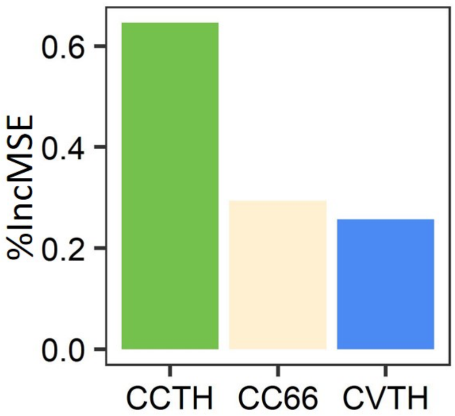

Supplementary Fig. 9 Relative importance of the three neighborhood canopy structural attributes to tree mortality rate during the 2012-2016 drought. Variable importance was assessed by the increase of mean-squared-error (%IncMSE) derived from a random forest regression analysis.

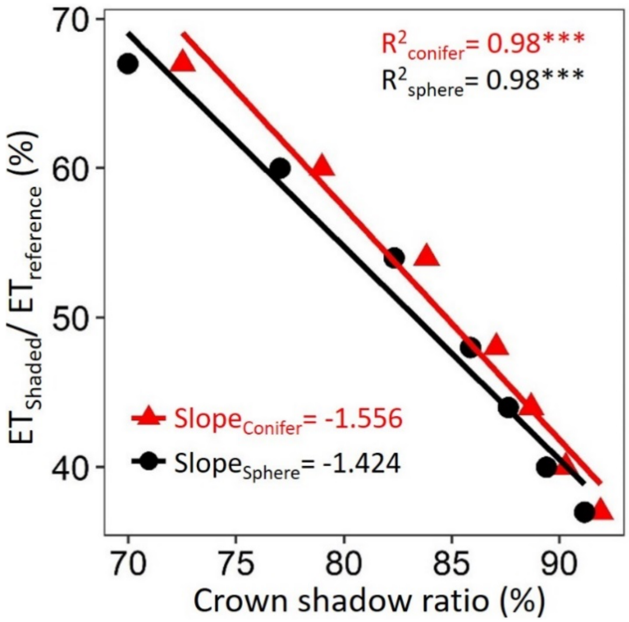

Supplementary Fig. 10 Responses of relative change in evapotranspiration (ET) to crown shadow ratio. The relative change in ET was calculated as the ratio between ET (ET_{shaded}) of a conifer-shaped or sphere-shaped tree under various crown shadow ratios (ranging from 73% to 92% with an interval of 3%) to that ($ET_{reference}$) of a conifer-shaped or sphere-shaped tree without crown shadow during the daytime (from 7:00 am to 6:30 pm) for a typical summer day (August 1st, 2016) in the study area. All ET values were simulated using the Boreal Ecosystem Productivity Simulator (see Supplementary Methods for details). The red triangles represent the simulation results of a conifer-shaped tree, and the black dots represent the simulation results of a sphere-shaped tree. The solid lines represent the fitted lines with a $P < 0.001$ (denoted as ***), and the coefficient of determination (R^2) and slope values are reported.

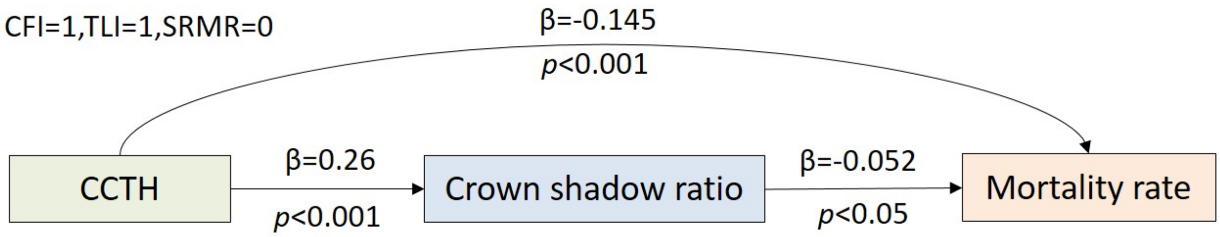

Supplementary Fig. 11 Pathways linking CCTH to tree mortality rate during drought through the regulation of crown shadow ratio derived from a structural equation modeling analysis. The structural equation modeling analysis was performed using R through the *lavaan* package. The coefficient (β) and *P* of each path are displayed, and the comparative fit index (CFI), Tucker-Lewis index (TLI), and standardized root mean square residual (SRMR) of the model are reported.

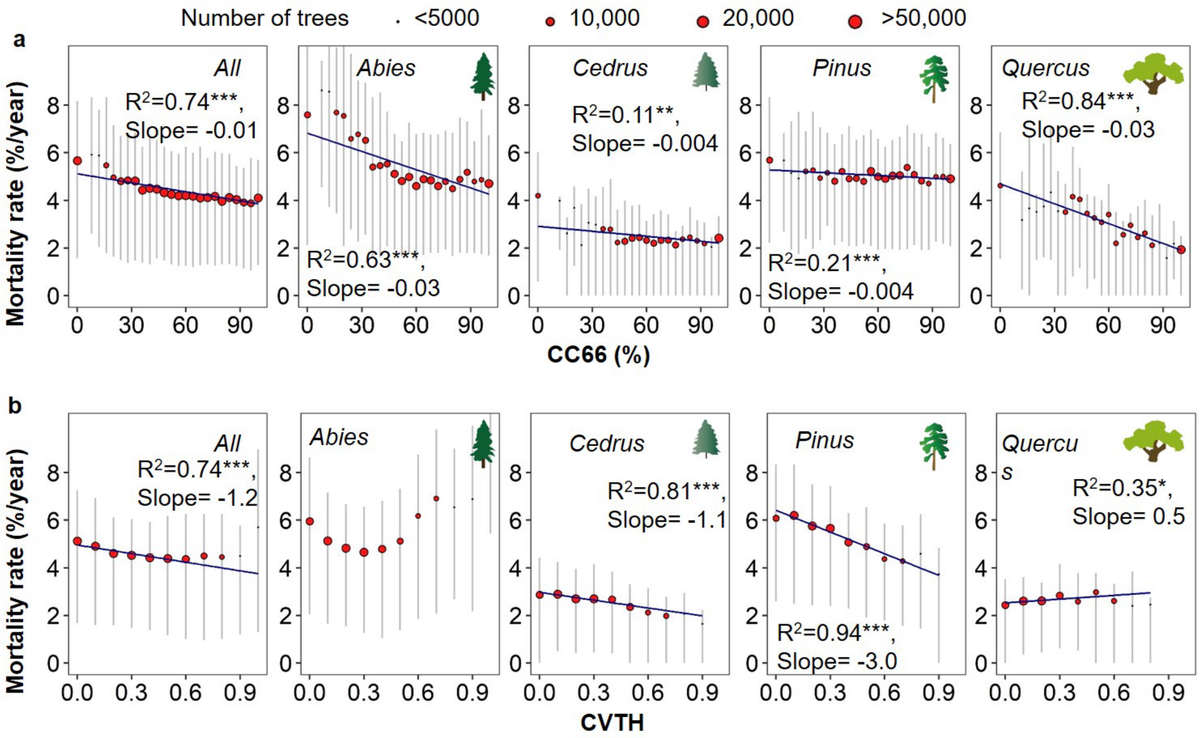

Supplementary Fig. 12 Relationships of tree mortality rate during the 2012-2016 drought with CC66 and CVTH. a, Relationships between CC66 and tree mortality rate of all genera (represented by panel *All*) and each respective genus. Forest stands were used as the basic statistical units here. Tree mortality rate within each forest stand was binned by CC66 with an interval of 2%, and the linear regression method weighted by the number of trees in each bin was used to fit their relationships. **b,** Relationships between CVTH and tree mortality rate of all genera (represented by panel *All*) and each respective genus. Forest stands were used as the basic statistical units here. Tree mortality rate within each forest stand was binned by CVTH with an interval of 0.1, and the linear regression method weighted by the number of trees in each bin was used to fit their relationships. Red dots in all panels represent the average tree mortality within each CC66 or CVTH bin, and grey lines represent the range of tree mortality rate between the first and third quartiles of each bin. Solid blue lines are fitted lines, and their R^2 and slope values are presented. *, **, and *** represent that the corresponding fitted line is significant at the confidence levels of 90%, 99%, and 99.9%, respectively.

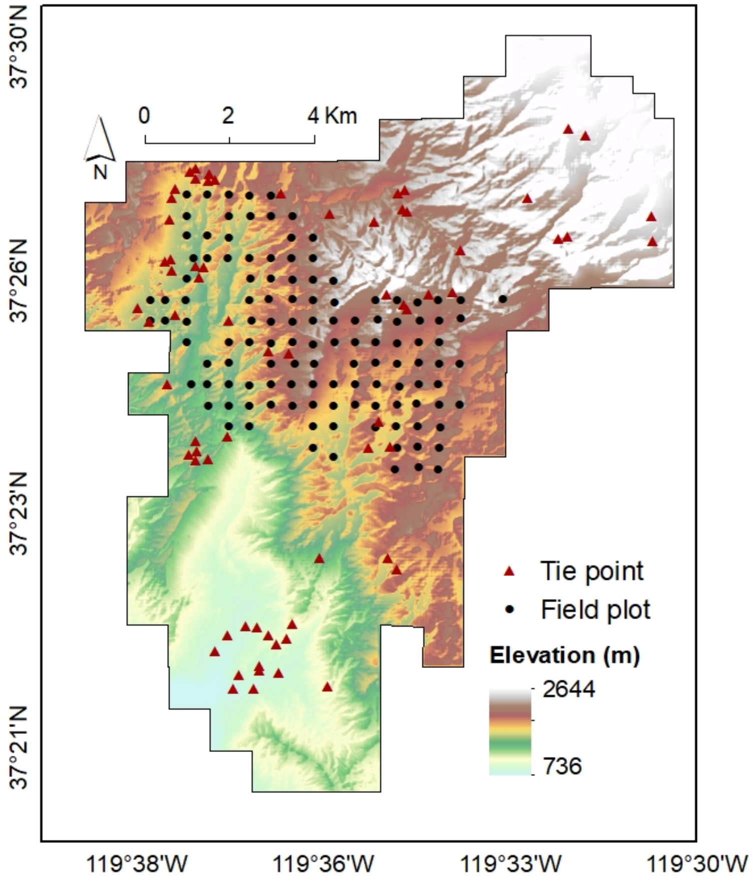

Supplementary Fig. 13 The distribution of field plot measurements and tie points within the study area. The tie points were used to geo-reference airborne lidar data and very-high-resolution aerial imagery. The background color shows its elevation distribution.

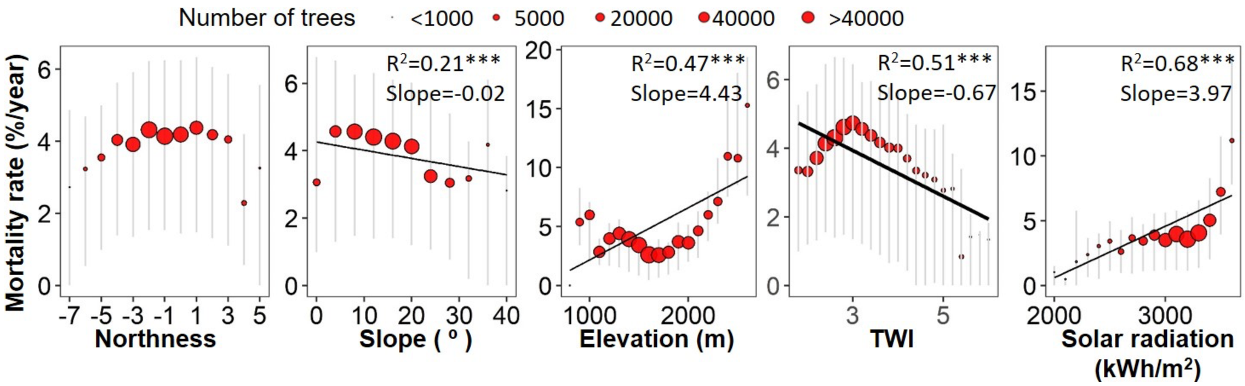

Supplementary Fig. 14 Relationships between environmental factors and tree mortality rate during the 2012-2016 drought. Five environmental factors were considered, including northness, slope, elevation, TWI and solar radiation. Forest stands were used as the basic statistical units here. Tree mortality rate within each forest stand was binned by northness (with an interval of 1), slope (with an interval of 5°), elevation (with an interval of 100 m), TWI (with an interval of 0.2), or solar radiation (with an interval of 100 Wh/m^2). The linear regression method weighted by the number of trees in each bin was used to fit their relationships. Red dots in all panels represent the average tree mortality within each bin, and grey lines represent the range of tree mortality rate between the first and third quartiles of each bin. Solid blue lines are fitted lines, and their R^2 and slope values are presented. *** represent that the corresponding fitted line is significant at a 99.9% confidence level.

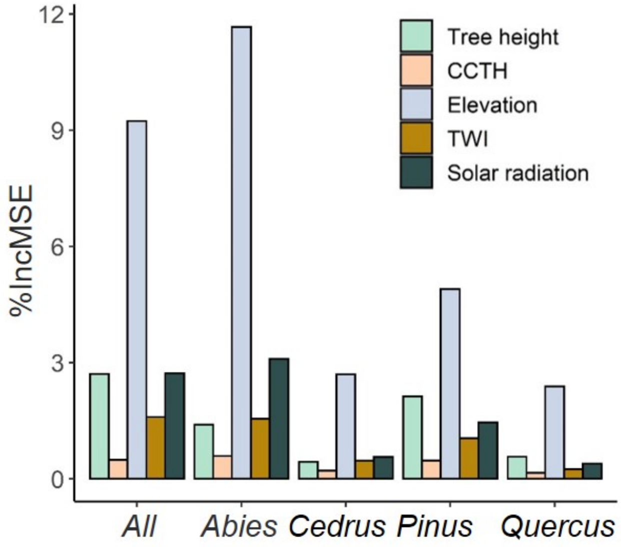

Supplementary Fig. 15 Relative importance of canopy structural attributes and environmental factors to tree mortality rate during the 2012-2016 drought. The relative importance is represented by %IncMSE derived from random forest regression analyses.

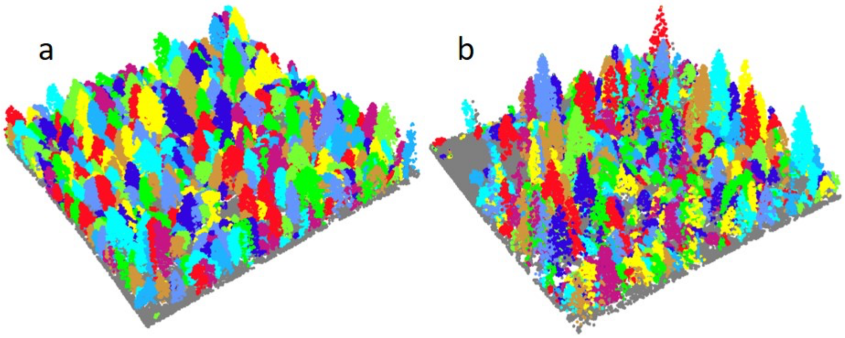

Supplementary Fig. 16. Examples of segmented individual trees. a, A randomly selected area with a relatively high tree density. **b,** A randomly selected area with a relatively low tree density. Each area has a size of 150 m × 150 m, and each cluster of points in different colors represents an individual tree.

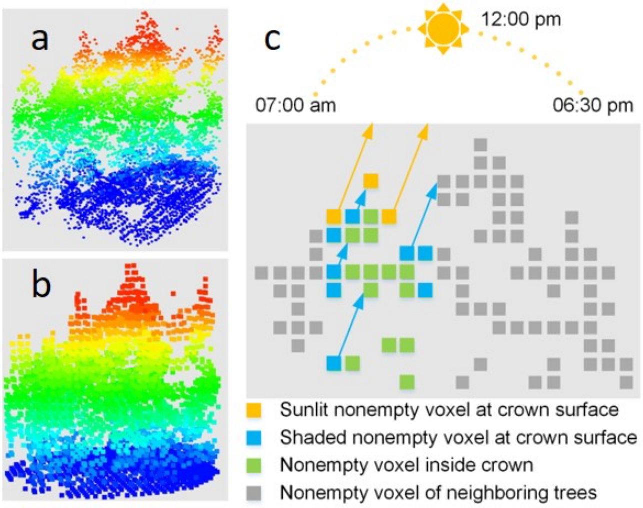

Supplementary Fig. 17 A conceptual diagram illustrating the simulation process of crown shadow. a, The original point cloud of a tree within its neighborhood (a circular buffer of 15 m in radius). **b,** The voxelized point cloud of the neighborhood. **c,** An illustration of the ray tracing-based shaded canopy simulation procedure.
