# Supplementary material for: Tree mortality during long-term droughts is lower in structurally complex forest stands
Source: Nat Commun. 2023 Nov 17;14:7467. doi: 10.1038/s41467-023-43083-8 (PMC10656564; doi:10.1038/s41467-023-43083-8)
Supplement: Supplementary file 3 — Reporting Summary [file 41467_2023_43083_MOESM3_ESM.pdf]

nature portfolio

Corresponding author(s): Yanjun Su

Last updated by author(s): Oct 31, 2023

Reporting Summary

Nature Portfolio wishes to improve the reproducibility of the work that we publish. This form provides structure for consistency and transparency in reporting. For further information on Nature Portfolio policies, see our Editorial Policies and the Editorial Policy Checklist.

Statistics

For all statistical analyses, confirm that the following items are present in the figure legend, table legend, main text, or Methods section.

n/a	Confirmed	
☐	☒	The exact sample size (*n*) for each experimental group/condition, given as a discrete number and unit of measurement
☒	☐	A statement on whether measurements were taken from distinct samples or whether the same sample was measured repeatedly
☐	☒	The statistical test(s) used AND whether they are one- or two-sided<br>*Only common tests should be described solely by name; describe more complex techniques in the Methods section.*
☐	☒	A description of all covariates tested
☐	☒	A description of any assumptions or corrections, such as tests of normality and adjustment for multiple comparisons
☐	☒	A full description of the statistical parameters including central tendency (e.g. means) or other basic estimates (e.g. regression coefficient) AND variation (e.g. standard deviation) or associated estimates of uncertainty (e.g. confidence intervals)
☐	☒	For null hypothesis testing, the test statistic (e.g. *F*, *t*, *r*) with confidence intervals, effect sizes, degrees of freedom and *P* value noted<br>*Give P values as exact values whenever suitable.*
☒	☐	For Bayesian analysis, information on the choice of priors and Markov chain Monte Carlo settings
☐	☒	For hierarchical and complex designs, identification of the appropriate level for tests and full reporting of outcomes
☐	☒	Estimates of effect sizes (e.g. Cohen's *d*, Pearson's *r*), indicating how they were calculated

Our web collection on statistics for biologists contains articles on many of the points above.

Software and code

Policy information about availability of computer code

Data collection	The field measurements were manually recorded. Lidar data were provided by the National Center for Airborne Laser Mapping. All other data used in the current study were downloaded from the web using a web browser, with detailed sources described in the Materials and Methods section.
Data analysis	Data analyses were performed using LiDAR360 version 5.2 (GreenValley International), ArcGIS version 10.8 (ESRI), SAGA-GIS version 7.9.1, and R version 4.2.1 . Details were reported in the Method section. The complete R code used for the calculation and visualization of the results is accessible on Figshare (doi: 10.6084/m9.figshare.24278014).

For manuscripts utilizing custom algorithms or software that are central to the research but not yet described in published literature, software must be made available to editors and reviewers. We strongly encourage code deposition in a community repository (e.g. GitHub). See the Nature Portfolio guidelines for submitting code & software for further information.

Data

Policy information about availability of data

All manuscripts must include a data availability statement. This statement should provide the following information, where applicable:

- Accession codes, unique identifiers, or web links for publicly available datasets
- A description of any restrictions on data availability
- For clinical datasets or third party data, please ensure that the statement adheres to our policy

The generated individual tree data with attributes of spatial locations, species, canopy structure, and live/dead conditions, as well as the processed data, are accessible on Figshare (doi: 10.6084/m9.figshare.24278014).

Research involving human participants, their data, or biological material

Policy information about studies with human participants or human data. See also policy information about sex, gender (identity/presentation), and sexual orientation and race, ethnicity and racism.

Reporting on sex and gender	N/A
Reporting on race, ethnicity, or other socially relevant groupings	N/A
Population characteristics	N/A
Recruitment	N/A
Ethics oversight	N/A

Note that full information on the approval of the study protocol must also be provided in the manuscript.

Field-specific reporting

Please select the one below that is the best fit for your research. If you are not sure, read the appropriate sections before making your selection.

☐ Life sciences ☐ Behavioural & social sciences ☒ Ecological, evolutionary & environmental sciences

For a reference copy of the document with all sections, see nature.com/documents/nr-reporting-summary-flat.pdf

Ecological, evolutionary & environmental sciences study design

All studies must disclose on these points even when the disclosure is negative.

Study description	We studied the influence of canopy structure on tree mortality in the southern Sierra Nevada during the 2012-2016 California drought. We identified nearly 1.5 million individual trees along with their species, structural features, and health condition during the drought using remote sensing datasets and field measurements. Field measurements were set up following a stratified sampling approach aligned with established practices in forest inventory. Our analyses showed trees overshadowed by tall neighboring trees experienced lower mortality rate, which highlighted that re-establishing heterogeneity in forest structure could improve forest resiliency to drought.
Research sample	We identified nearly 1.5 million trees over the study area using light detection and ranging (lidar) data collected in 2012. Tree species were classified into four genera (Abies, Cedrus, Pinus, and Quercus) based on very-high resolution (VHR) aerial imagery and 121 field plots surveyed in the summer of 2007 and 2008. Dead trees were mapped from time-series VHR aerial imagery collected in 2012, 2014, and 2016.
Sampling strategy	We used all tree samples identified from remote sensing and field measurements. Details were provided in the Methods section and the Supplementary Materials.
Data collection	The airborne lidar data were collected in the summer of 2012 using an Optech GEMINI airborne laser terrain mapper system, which was flown at a height of 600 - 1000 m above the ground. The high-resolution aerial images were collected by the National Agriculture Imagery Program (NAIP) in 2012, 2014, and 2016, respectively. Field plots for tree species mapping were collected by the University of California team of SNAMP. Their locations and tree hegihts were measured using a Trimble GeoXH Global Positioning System (GPS) unit equipped with a Trimble Zephyr antenna, an Impulse laser ranger finder, and an Impulse electronic compass.
Timing and spatial scale	This study was conducted for the time of California drought, i.e., 2012-2016, and the study area was located in the southern Sierra Nevada with a size of approximately 151 km2. Lidar data were collected in the year of 2012, and VHR imagery were collected in 2012, 2014, and 2016, respectively. All remote sensing data covered the entire study area. A total of 121 field plots (12.62 m in diameter) were collected in the summer of 2007 and 2008. Details were provided in the Methods section.

Data exclusions	We only analyzed tree samples with a height taller than 5 m, because small trees might be difficult to be identified from aerial imagery.
Reproducibility	The results are fully reproducible using the data and methods detailed in the manuscript.
Randomization	We used random forest models to map tree species groups and dead trees. A half of samples were randomly selected for training, and the remaining samples were used as validation.
Blinding	There was no blinding applicable in this study.

Did the study involve field work? ☒ Yes ☐ No

Field work, collection and transport

Field conditions	The field plots were measured during the summer of 2007 and 2008. There was no rainfall, the temperature was between 20 to 35 Celsius degrees during the field work.
Location	The field site is located in the southern Sierra Nevada mountainous forests (37°25′N, 119°36′W), and its elevation ranging from 730 m to 2650 m above sea level.
Access & import/export	The field work only recorded tree location, size, and species measurements. No samples were collected from the field. Therefore, no permit was required.
Disturbance	There was almost no disturbance during the field work. Field crews walked carefully to sites to avoid disturbances to the field.

Reporting for specific materials, systems and methods

We require information from authors about some types of materials, experimental systems and methods used in many studies. Here, indicate whether each material, system or method listed is relevant to your study. If you are not sure if a list item applies to your research, read the appropriate section before selecting a response.

Materials & experimental systems

n/a	Involved in the study	
☒	☐	Antibodies
☒	☐	Eukaryotic cell lines
☒	☐	Palaeontology and archaeology
☒	☐	Animals and other organisms
☒	☐	Clinical data
☒	☐	Dual use research of concern
☒	☐	Plants

Methods

n/a	Involved in the study	
☒	☐	ChIP-seq
☒	☐	Flow cytometry
☒	☐	MRI-based neuroimaging
